# Validation of a Spanish version of the Spine Functional Index

**DOI:** 10.1186/1477-7525-12-96

**Published:** 2014-06-27

**Authors:** Antonio I Cuesta-Vargas, Charles P Gabel

**Affiliations:** 1 Universidad de Malaga, Andalucia Tech, Facultad de Ciencias de la Salud, Departamento de Psiquiatria y Fisioterapia, Instituto de Biomedicina de Malaga (IBIMA), Grupo de Clinimetria (AE-14), Av/ Arquitecto Peñalosa s/n (Teatinos Campus Expansion), Malaga, Spain; 2 School of Clinical Science, Faculty of Health at the Queensland University of Technology, Brisbane, Australia; 3 Centre for Healthy Activities, Sport and Exercise of the Faculty of Science at the University of the Sunshine Coast Queensland, Brisbane, Australia

**Keywords:** Spine, Psychometrics, Outcome measure, Spanish

## Abstract

**Background:**

The Spine Functional Index (SFI) is a recently published, robust and clinimetrically valid patient reported outcome measure.

**Objectives:**

The purpose of this study was the adaptation and validation of a Spanish-version (SFI-Sp) with cultural and linguistic equivalence.

**Methods:**

A two stage observational study was conducted. The SFI was cross-culturally adapted to Spanish through double forward and backward translation then validated for its psychometric characteristics. Participants (n = 226) with various spine conditions of >12 weeks duration completed the SFI-Sp and a region specific measure: for the back, the Roland Morris Questionnaire (RMQ) and Backache Index (BADIX); for the neck, the Neck Disability Index (NDI); for general health the EQ-5D and SF-12. The full sample was employed to determine internal consistency, concurrent criterion validity by region and health, construct validity and factor structure. A subgroup (n = 51) was used to determine reliability at seven days.

**Results:**

The SFI-Sp demonstrated high internal consistency (α = 0.85) and reliability (r = 0.96). The factor structure was one-dimensional and supported construct validity. Criterion specific validity for function was high with the RMQ (r = 0.79), moderate with the BADIX (r = 0.59) and low with the NDI (r = 0.46). For general health it was low with the EQ-5D and inversely correlated (r = −0.42) and fair with the Physical and Mental Components of the SF-12 and inversely correlated (r = −0.56 and r = −0.48), respectively. The study limitations included the lack of longitudinal data regarding other psychometric properties, specifically responsiveness.

**Conclusions:**

The SFI-Sp was demonstrated as a valid and reliable spine-regional outcome measure. The psychometric properties were comparable to and supported those of the English-version, however further longitudinal investigations are required.

## Introduction

Patient reported outcome (PRO) measures [1,2] are a required and integral part of the patient health management process. The PROs provide objective responses on status and function that assist clinicians, surgeons and researchers to track a patients progress and determine if status has changed. These changes, or the lack, can be a consequence of natural healing or an intervention, be that conservative or surgical [3]. This external quantification process has been progressively adopted and accurately reflects the patient’s health status by means of a self-report methodology. This process has progressively superseded the traditional model of therapist determined clinical signs and symptoms and generic quality of life measures. In this way the clinicians' and researchers’ understanding of how the patient’s function and symptoms have changed, over time or in response to an intervention, can be rapidly assimilated. This is applicable for a wide range of conditions, diseases and injuries and assists the progressive management through recognition of the effects on the patient's capabilities [4]. As this patient focused paradigm of management has been adopted and progressed in musculoskeletal medicine over the last two decades, there has been a gradual shift from condition or disease specific measures towards the use of region specific PROs. These regional tools reflect the status and any changes within the three key kinetic-chain regions of the upper limb [5], lower [6] limb and spine [7]. Consequently they are adopted more frequently as the standard protocol for measurement and assessment of functional status [8].

The Spine Functional Index (SFI) is a recently proposed whole-spine regional PRO. Published in 2013, the SFI was shown to have strong clinimetric properties for both the psychometric and practical characteristics [7]. These included reliability, validity, responsiveness, error measurement and internal consistency as well as brevity, rapid transfer to a 100-point or percentage scale, ease and brevity for completion, low missing responses, suitable readability and a single factor structure [7] that enables summation to a single unique score [9]. The findings also showed preferable clinimetric properties to the Functional Rating Index [10] for the whole spine [7]. The translation to a Spanish version was warranted as it would support the comparable findings for the functional index series that include the upper limb [5] and lower limb [7], each of which was found to be preferable to recognized and advocated English criterion PROs both within the original development studies and within independent research that included Spanish and other language translated versions [11-13].

A Spanish version of the SFI was not yet developed or validated. Given that Spanish is one of the five most spoken languages and the world’s second widest geographically spoken language [13], it would seem appropriate for a SFI Spanish version (SFI-Sp) to be developed to meet this need. Consequently the aims of this paper were: to describe the translation and cross-cultural adaptation process of the English SFI version to Spanish; and to assess for clinical use with Spanish speakers the critical psychometric properties of reliability, factor structure, internal consistency and concurrent criterion validity. An *a-priori* hypothesis for criterion validity was that it would be high to moderate for back and neck region specific PROs and low to moderate and inversely related to general health PROs or their subcategory components.

## Materials and methods

### Design

A two-stage observational study design was employed. Stage 1 involved the initial Spanish translation and cross-cultural adaptation of the SFI [7]. Stage 2 involved prospective evaluation of the SFI-Sp’s four critical psychometric properties through concurrent completion in a physical therapy outpatients’ setting.

All study participants completed five questionnaires. These included two generic health measures, the SFI and a regional specific PRO for the neck or back depending on the patients diagnosis and primary symptomatic region, For the neck a single PRO was used - the Neck Disability Index (NDI) [14] while for the back two PROs were concurrently employed - the Roland Morris Questionnaire (RMQ) [15] and the Backache Index (BADIX) [16]. The approach enabled a criterion specific comparison for the whole-spine by region, while clarification and criterion comparison of the participants’ health status was provided by the EuroQol Health Questionnaire 5 Dimensions (EQ-5D) [17] and the Short Form twelve (SF-12) [18]. Two assessors performed all initial and subsequent assessments but were blinded to baseline scores to ensure independent collection of outcome data.

### Stage 1 - translation of the SFI to the “SFI-Sp”

**Figure 1:**
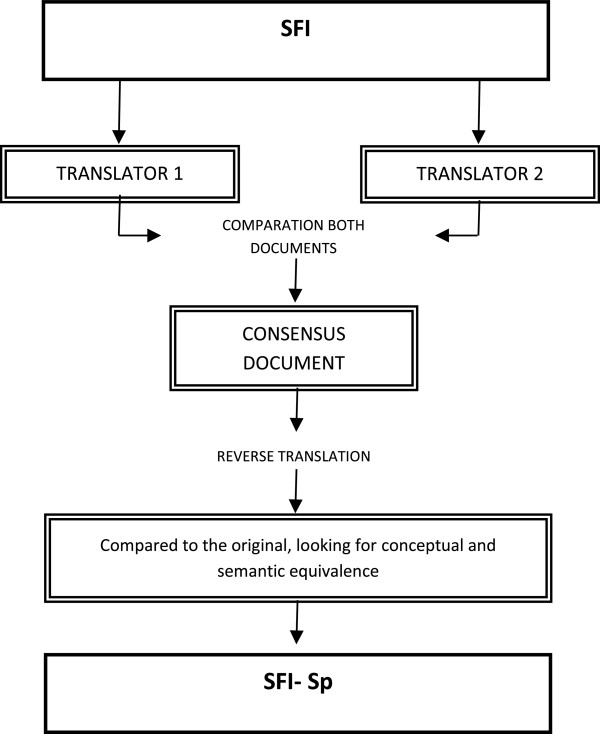
Flowchart of the translation of the Spine Functional Index (SFI) from English to Spanish.

The primary objective of Stage 1 was to ensure that the Spanish translation was conceptually equivalent to the original English version. A forward and backward translation methodology was applied that involved two specialist in the field of translation, for each directional process as detailed and recommended in the specialized scientific literature (Figure 1) [11,19,20]. Completion of the back-translation resulted in draft formats with a final consensus version gained from both translators. The differences in versions were minimal and this consensus version was achieved with unanimity and without difficulty.

### Stage 2 - prospective psychometric investigation

#### Participants, setting and procedure

A total of 226 consecutive volunteers (48 ± 19 years, 54.4% female) were diagnosed by a general practitioner (GP) with non-specific low back pain of a mechanical and degenerative nature using Waddell’ s classification for acute and chronic conditions [21]. All participants were then referred to two Spanish physiotherapy outpatient clinics. Exclusion criteria were refusal to participate in the study, low back pain (LBP) as a result of a specific spinal disease, infection, presence of a tumor, osteoporosis, fracture, structural deformity, inflammatory, disorder, radicular symptoms or cauda equina syndrome.

The inclusion criteria were a neck or back injury of a mechanical or degenerative nature as diagnosed by a medical practitioner. The presenting conditions and diagnoses were broadly classified into four regional sub-categories (Table 1). The exclusion criteria were age <18 years and poor Spanish language comprehension as required for the completion of the questionnaires.

**Table 1:** Demographic characteristics and frequency of diagnosis of the study population

***Characteristic***	***Cases (%)***	***Age (years) Mean (sd)***
*Study Population*	226	45 ± 7
Male	95 (42%)	47 ± 6
Female	131 (58%)	46 ± 8
*Sub-region*		
Cervical	93 (41%)	46 ± 4
Thoracic	9 (4%)	39 ± 6
Lumbar	111 (49%)	50 ± 4
Multi-area	13 (6%)	46 ± 6

The full sample (n = 226) completed the EQ-5D, SF12 and SFI-Sp questionnaires. The neck and multi-area participants completed the NDI (n = 106; 93 + 13).

The lumbar, thoracic and multi-area participants completed the RMQ and BADIX (n = 133; 111 + 9 + 13).

### Spine functional index (SFI)

The *SFI* is a 25-item regional PRO with a 3-point response option of ‘Yes’ , ‘Partly’ and No’ that requires around one minute to complete. The score is calculated from simple addition of the responses then multiplied by four to provide a percentage scale and subtracted from 100 to give a functional score relative to the patients’ pre-injury or normal status. Up to two missing responses are permitted [7].

### Neck disabilities index (NDI)

The NDI is a ten-item questionnaire that requires the user to select one of six statements per item question that best describes their individual status at that time [14,22]. According to Young et al. [23]. the NDI is a PRO scale dealing with impairments in bodily function (i.e., reading, concentration) that can be considered as psychological constructs [24]. It also considers items dealing with physical limitations of function (i.e., lifting, driving) [12]. Each question-item has six potential responses ranging in severity from zero (no disability) to five (most severe disability) with a maximum total score of 50 points. This is subsequently multiplied by two to provide a percentage scale where 100% indicates most severe disability, 0% indicates no disability. The cut-off scores for the NDI are recognised as ≤8 NDI-points reflects no disability or recovered and >28 NDI-points indicates moderate to severe disability or severity [14,21].

### Roland Morris questionnaire (RMQ)

The RMQ is a 24 item back-specific scale derived from the Sickness Impact Profile [23] by addition of the phrase “because of my back”. Each item is answered “yes” or “no” where each positive response is scored as 1 and each negative response (question without mark) is scored 0. This yields a final score ranging from 0 (no disability) to 24 (maximum disability). The reliability of the Spanish version is reported at CCI =0.87 [13].

### Backache disability index (BADIX)

The “Backache Disability Index” for LBP or BADIX includes a rating of 5 trunk movements in the erect position resulting in a “Backache Index (BAI)”, and one “Morning Back Stiffness (MBS)” score. The sum of the BAI and MBS gives the BADIX (max. 20 points) [16].

The BAI consists of one flexion test, lateral flexion bilaterally and extension combined with both sides of lateral flexion. The results are recorded on a specific form on which the 4-point score per outcome is indicated. The observer notes the scoring outcomes (points) and the sum of the five outcomes yields the BAI with a maximum of 15 points [19]. Reliability coefficients of the Spanish version of the BAI are reported at 0.97 [19].

The MBS is determined by asking the patient what phrase corresponds best to their feeling or concern about their LBP following a minimum of 6 hours sleep. There are six response options scored on a 0–5 point scale where 0 = no MBS and 5 = high MBS. This is reported as “I can/cannot (need help) to stand up from my bed without/with restriction and I feel no/only irritation/pain/much pain in my back”. The observer notes the score (points) and the sum of the five outcomes yields the MBS with a maximum of five points.

### Euroqol health questionnaire 5 dimensions (EQ-5D)

The EQ-5D-3 L is a widely used six-item non-disease-specific questionnaire that has been demonstrated as valid and reliable in the Spanish population [17]. It has five 3-point response options for different quality-of-life dimensions and a sixth question on overall perceived health-related status on a 100 mm Visual Analogue Scale (VAS). The EQ-5D-3 L-VAS reflects the respondent’s self-rated health status and is ranked from ‘Best Imaginable’ (100) to Worst Imaginable’ (0).

### Short form health status survey (SF-12)

The SF-12 is a PRO that estimates the general health state of a person based on two components: physical and mental (SF-12 PCS and SF-12 MCS). In English speaking countries the reliability of the SF-12 PCS was reported between 0.86 and 0.89 and for the SF-12 MCS between 0.76 and 0.77 [18].

### Statistics

*Descriptive analyses* were applied to calculate means and standard deviations of the demographic variables (Table 1). *Distribution and normality* were determined by the one-sample Kolmogorov-Smirnov tests (significance >0.05). *Construct validity and factor structure* were determined from maximum likelihood extraction (MLE) with the *a-priori* extraction requirements being satisfaction of three criteria: screeplot inflection, Eigenvalue >1.0 and variance >10%. The recommended minimum ratio of five participants-per-item was satisfied [25]. *Exploratory factor analysis* indicated a single factor structure was likely, therefore more 100 participants were required [26]. The *internal consistency* was determined from Cronbach's α coefficient as calculated at an anticipated value range of 0.80-0.95 [27]. It was hypothesized that there would be no difference in the mean item scores between male and female participants. The mean scores were compared using a Student’s t-test.

*Criterion validity* was determined through the concurrent use of all PRO measures (NDI, RMQ, BADIX, EQ-5D, SF12 and SFI-Sp). The Pearson’s r correlation coefficient used the criteria of poor (r ≤ 0.49), fair (r = 0.50-0.74) and strong (r ≥ 0.75) [28].

*Reliability* was performed using the Intraclass Correlation Coefficient Type 2,1 (ICC_2.1_) test-retest methodology in a randomly selected subgroup of the full sample (n = 45, 49 ± 3 years, 56.1% female) recorded at baseline and one week (seven days). The sub-groups presenting conditions were representative of the four sub-categories of the full sample using scores on the SFI-Sp.

The *sensitivity or error score* was determined from the minimum detectable change (*MDC*_*90*_*)* analysis that was performed as described by Stratford [29]. The standard error of the measurement (SEM) was calculated using the formula: SEM = s√(1–r), where s = the mean and standard deviation (SD) of time 1 and time 2, r = the reliability coefficient for the test and Pearson’s correlation coefficient between test and retest values. Thereafter the MDC_90_ was calculated using the formula: MDC_90_ = SEM × √2 × 1.65.

The minimum *sample sizes* for the validation study were calculated from the original study for an 80% likelihood of detecting differences allowing for 15% attrition with p < .05 [28]. Power calculations indicated the need for a minimum sample of n ≥ 110 (reliability, n ≥ 45; and concurrent criterion validity, n ≥ 106) [28].

All *statistical analyses* were conducted using the Statistical Package for Social Science version 17.0 (SPSS 17.0) for Windows and LISREL 8.80 [30].

The Tribunal of Review of Human Subjects at the University of Malaga approved ethical clearance.

## Results

### Characteristics descriptive of the participants

**Figure 2:**
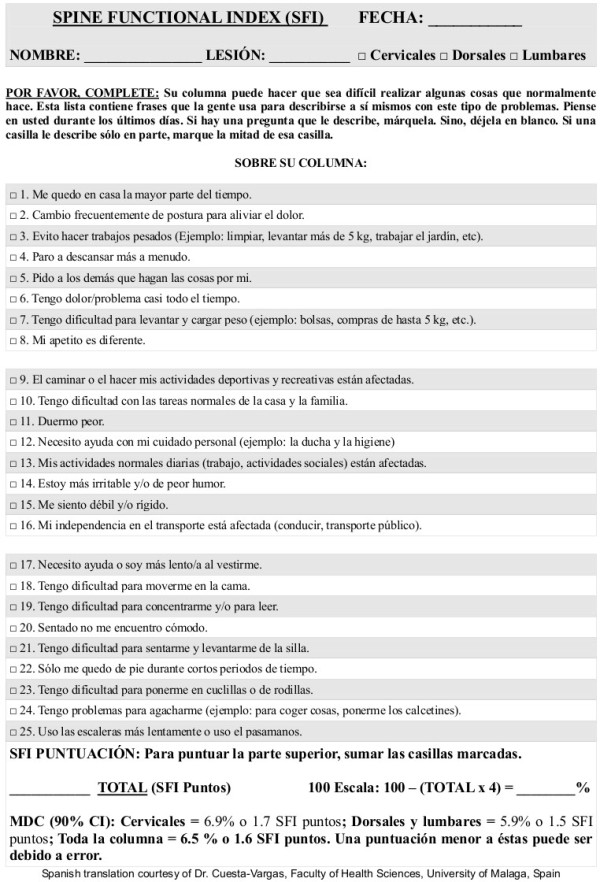
Spanish Spine Functional Index.

The demographic and frequency of diagnosis of the study sample are detailed in Table 1. The SFI was translated and back translated with consideration of the Spanish cultural linguistic adaptation to provide the new SFI-Sp questionnaire without language difficulties or other conceptual misunderstanding (Figure 2). The mean and standard deviation values for SFI-Sp score were determined (5.88 ± 5.6 points). There were no missing responses on the SFI-Sp and a high degree of *internal consistency* was observed (α = 0.845) with an individual item α range of 0.80 to 0.88. The *test-retest reliability* was high at (r = 0.96) with an individual range of 0.93 to 0.98. The total summated score was used in the analysis, not the scores of the individual questions. *Measurement error* from SEM and MDC90 were 2.81% and 6.89% respectively. No significant gender differences were found in the item responses.

**Figure 3:**
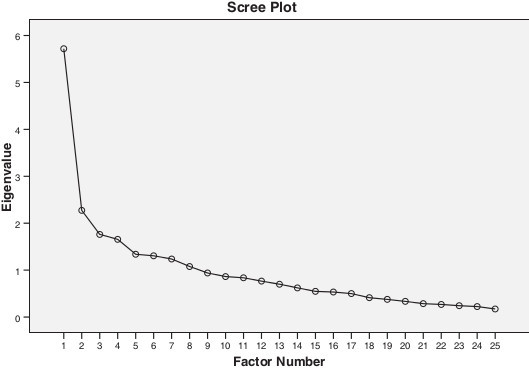
Scree Plot of the exploratory one-factor solution.

For *factor analysis* the correlation matrix for the LLFI-Sp was determined as suitable from the Kaiser-Meyer-Oklin values (0.826) and Barlett’s Test of Sphericity (p < 0.001). This indicated that the correlation matrix was unlikely to be an identity matrix and was therefore suitable for MLE. The screeplot (Figure 3) indicated a one-factor solution as determined by satisfaction of all three *a-priori* factors of the screeplot inflection point, an Eigenvalue > 1 and variance >10%. The factor analysis revealed a satisfactory percentage of total variance explained by the one factor at 27.4%. It was noted that eight factors had Eigenvalues >1.0 and accounted for 68.7% of variance; however those with an Eigenvalue >1.0 each accounted for <10% of variance and were shown to be after the screeplot initial inflection point (Figure 3) and consequently not extracted [31,32]. The items loading for the one-factor solution for the MLE method and average score for each item are shown in Table 2.

**Table 2:** Factor loading items for the one-factor solution, average score and discrimination indices of items

**Question**	**Item**	**Factor loading**	**Item average score**	**Item discr indices**
1	Stay at home most of time	,586	,09	,628(**)
2	Change positions frequently	,293	,66	,314(**)
3	Avoid heavy jobs	,314	,67	,336(**)
4	Rest more often	,521	,29	,559(**)
5	Get others to do things	,323	,07	,346(**)
6	Pain almost all the time	,570	,38	,610(**)
7	Lifting and carrying	,379	,59	,407(**)
8	Appetite affected	,127	,04	,136
9	Walking/normal recreation/sport	,512	,38	,549(**)
10	Home/family duties and chores	,604	,16	,647(**)
11	Sleep less well	,538	,37	,577(**)
12	Assistance with personal care, hygiene	,296	,02	,318(**)
13	Regular daily activity work/social	,602	,20	,318(**)
14	More irritable/bad tempered	,370	,25	,645(**)
15	Feel weaker or stiffer	,442	,44	,396(**)
16	Transport independence	,401	,06	,474(**)
17	I require assistance or am slower with dressing	,518	,13	,429(**)
18	I have difficulty moving in bed	,500	,14	,555(**)
19	I have difficulty concentrating and / or reading	,233	,21	,536(**)
20	My sitting is affected	,459	,14	,250(**)
21	I have difficulty getting in and out of chairs	,414	,35	,492(**)
22	I only stand for short periods of time	,256	,39	,443(**)
23	I have difficulty squatting and / or kneeling down	,472	,38	,275(**)
24	I have trouble reaching down (e.g. pick-up things, put on socks)	,430	,35	,506(**)
25	I go up stairs slower or use a rail	,538	,29	,461(**)

Symbol ** =significance values p<0.05.

Criterion specific validity with RMQ was high (r = 0.79), with BADIX and NDI was moderate (r = 0.59 and r = 0.46, respectively). Criterion standard validity with the EQ-5D was poor and inversely correlated (r = −0.42) and with Physical and Mental Component of SF-12 it was fair and inversely correlated (r = −0.56 and r = −0.48), respectively.

## Discussion

### Main findings

The SFI was translated to provide a cross-cultural adaptation to the Spanish language. The translation process ensured the conceptual equivalence of the used terms. This provided accessibility to the SFI for the world’s second largest geographically spoken language. The psychometric properties, specifically construct and criterion validity, reliability and internal consistency were determined independently and found to be strong and the single factor structure indicated a single summated score could be used [9].

The cross-cultural adaptation of the SFI into Spanish enables clinicians in Spanish speaking settings to compare outcomes following their treatments and interventions that affect the spine. The procedure of cross-cultural adaptation adopted for this study reflects that used in previous studies for different scales and applied in the Spanish context [19,20]. It is critical to employ research measures that are both culturally and linguistically appropriate if they are to be both valid and reliable [20].

The one-factor solution determined by the factor analysis accounted for a significant proportion of variance [31,32] and showed evidence that supports the presence of construct validity. A one-factor solution is critical if a PRO is to be used with a single summated score and subsequently reflect the construct for which it is primary used – that of representation of the functional status of the whole-spine [7].

The three other psychometric properties were also shown to be high and well supported. The internal consistency (α = 0.845) was lower but close to that of the original English version (0.91) [7], which sits below the accepted 0.95 thresholds for item redundancy [30]. The test-retest reliability or reproducibility (r = 0.96) was also equivalent to the original instrument (0.97) [7]. The criterion validity with the RMQ was demonstrated as strong and with BADIX and NDI was fair, suggesting transferability and substitution is a potential option. The EQ-5D-3 L being poor and inversely correlated and the Physical and Mental Components of SF-12 being fair and inversely correlated indicate that the SFI-Sp has limited value in indicating general health status.

The negative correlations support that deteriorating health was correlated with worsening function (higher scores on the SFI-Sp).

### Study strengths and limitations

The *strengths of the study* include the prospective nature and the adequate sample size that provided a suitable power for analysis for the sample as a whole-spine, single kinetic chain population [33]. The inclusion of consecutive patients, independence of the assessors and referral source, along with the broad diagnosis and category representations suggests limited selection bias and potential population generalizability [23]. The similarity in the psychometric properties between the English and Spanish SFI versions indicated a broad cross-cultural adaption may be appropriate. The SFI-Sp also has the potential to provide comparable whole-spine health status in Spanish-speaking patients with their English-speaking counterparts in countries with a high Spanish-speaking population such as the United States. However a direct population comparative study will be required and to determine if equivalent scores for patients with the same degree of injury severity have equivalent SFI scores.

The *study limitations* include the lack of longitudinal data regarding other psychometric properties, particularly responsiveness or sensitivity to change and error scores as a representation of a minimal clinically important difference. The determination of validity by diagnostic subgroup and sample was not possible as such sub allocation rendered the sample size insufficient for power analysis. An analysis by sub-region of back or neck was not performed as this would not reflect the whole-spine single kinetic chain. A potential limitation is that the participant patients were not involved in the translation process and developed of the tool. The determination of construct validity through the use of factor analysis represents only one possible statistical method of testing. A construct is not restricted to one set of observable indicators or attributes. There is a need for additional indicators in future research. Similarly, the practical characteristics were not determined. Finally, the inclusion of Hispanic/Latino/ South American participants in future studies could potentially provide confirming or conflicting linguistic information due to the cultural and ethnic difference with respect to the Spanish participants and their cultural diversity in terms of European versus the Americas, North, Central and South.

## Conclusions

The SFI is translated and cross-culturally adapted to Spanish for the first time. The psychometric properties of this SFI Spanish-version are also reported with the determined values found to be satisfactory and supportive of the findings of the SFI scale in the English format, particularly in the areas of internal consistency, factor structure and reliability. Consequently the SFI-Sp may be useful in Spanish-speaking populations and for use in cross-ethnic and cross-cultural comparisons in other English speaking countries with a high Spanish-speaking population. There will be a need for further research to determine if this PRO is influenced by the type of spine pathology or specific subgroups of patients.

## Competing interests

No competing financial interests exist.

## Authors’ contributions

All the authors have made contributions to conception of this study. Antonio I. Cuesta-Vargas and Phillip C Gabel participated in the analysis and interpretation of data and were involved in drafting the manuscript or revising it critically for important intellectual content. All the authors have given final approval of the version to be published.
